# Host immune responses induced by specific *Mycobacterium leprae* antigens in an overnight whole-blood assay correlate with the diagnosis of paucibacillary leprosy patients in China

**DOI:** 10.1371/journal.pntd.0007318

**Published:** 2019-04-24

**Authors:** Xiaohua Chen, Yuan-Gang You, You-Hua Yuan, Lian C. Yuan, Yan Wen

**Affiliations:** 1 Beijing Tropical Medicine Research Institute, Beijing Friendship Hospital, Capital Medical University, Beijing, China; 2 Beijing Key Laboratory for Research on Prevention and Treatment of Tropical Diseases, Capital Medical University, Beijing, China; 3 Department of Laboratory, Henan Provincial People’s Hospital, Zhengzhou, Henan, China; Hospital Infantil de Mexico Federico Gomez, UNITED STATES

## Abstract

**Background:**

Leprosy, caused by *Mycobacterium leprae*, affects over 200,000 people annually worldwide and remains endemic in the ethnically diverse, mountainous and underdeveloped southwestern provinces of China. Delayed diagnosis of leprosy persists in China, thus, additional knowledge to support early diagnosis, especially early diagnosis of paucibacillary (PB) patients, based on the host immune responses induced by specific *M*. *leprae* antigens is needed. The current study aimed to investigate leprosy patients and controls in Southwest China by comparing supernatants after stimulation with specific *M*. *leprae* antigens in an overnight whole-blood assay (WBA) to determine whether host markers induced by specific *M*. *leprae* antigens improve the diagnosis or discrimination of PB patients with leprosy.

**Methodology/Principal findings:**

Leprosy patients [13 multibacillary (MB) patients and 7 PB patients] and nonleprosy controls [21 healthy household contacts (HHCs), 20 endemic controls (ECs) and 19 tuberculosis (TB) patients] were enrolled in this study. The supernatant levels of ten host markers stimulated by specific *M*. *leprae* antigens were evaluated by overnight WBA and multiplex Luminex assays. The diagnostic value in PB patients and ECs and the discriminatory value between PB patients and HHCs or TB patients were evaluated by receiver operator characteristics (ROC) analysis. ML2044-stimulated CXCL8/IL-8 achieved the highest sensitivity of 100%, with a specificity of 73.68%, for PB diagnosis. Compared to single markers, a 3-marker combination model that included ML2044-induced CXCL8/IL-8, CCL4/MIP-1 beta, and IL-6 improved the diagnostic specificity to 94.7% for PB patients. ML2044-stimulated IL-4 and CXCL8/IL-8 achieved the highest sensitivity (85.71% and 100%) and the highest specificity (95.24% and 84.21%) for discriminating PB patients from HHCs and TB patients, respectively.

**Conclusions:**

Our findings suggest that the host markers induced by specific *M*. *leprae* antigens in an overnight WBA increase diagnostic and discriminatory value in PB patients with leprosy, with a particularly strong association with interleukin 8.

## Author summary

Leprosy, caused by *Mycobacterium leprae*, affects over 200,000 people annually worldwide and remains endemic in the ethnically diverse, mountainous and underdeveloped southwestern regions of China. Although it is curable, delayed diagnosis of leprosy persists in China, with a disability rate as high as 20% nationwide. To identify the source of infection and block transmission more effectively, further knowledge about the diagnostic value of antigen-specific induced host immune responses in patients is needed. The current study aimed to evaluate the diagnostic value of an overnight whole-blood assay for the diagnosis of paucibacillary (PB) leprosy patients, and differences in the ability of specific *M*. *leprae* antigens to stimulate a panel of host markers was tested by an overnight whole-blood assay. Our findings suggest that host markers induced by specific *M*. *leprae* antigens in an overnight WBA have diagnostic value in leprosy patients and discriminatory value between leprosy patients and healthy household contacts (HHCs) or tuberculosis (TB) patients.

## Introduction

Leprosy is a treatable infection that is caused by *Mycobacterium leprae* (*M*. *leprae*) and can result in skin lesions, nerve degeneration, and deformities. The current World Health Organization (WHO) directives for leprosy control programs encourage widespread administration of multidrug therapy (MDT) to treat patients and early diagnosis [1]. The implementation of WHO MDT treatment has drastically reduced the number of registered leprosy cases from the approximately 12 million reported cases in 1985 to fewer than 250,000 reported cases in 2006 [2]. In 2017, 210,671 new cases of leprosy were detected, and the registered prevalence was 192,713 cases [3]. Indeed, assays for detecting *M*. *leprae*-specific IgM antibodies against PGL-I have been developed successfully [4,5], and these assays are able to identify multibacillary (MB) leprosy patients (with strong humoral immunity against *M*. *leprae*) [6]. However, as only 1–5 skin lesions, 1–2 damaged nerves, and few bacteria are present, the diagnosis and discrimination of paucibacillary (PB) patients with leprosy remain challenging. Therefore, antigen-specific immune diagnostic tools, especially antigen-specific secretion of host markers in whole-blood assays (WBAs), have been an important topic in leprosy research.

Commercially available IFN-gamma (IFN-γ) release assays (IGRAs) such as Quanti-FERON-TB Gold have been developed successfully for specific detection of *M*. *tuberculosis* infection and discrimination from all (nonvirulent) BCG strains and most other nontuberculous mycobacteria (NTMs) [7], which has inspired research into the feasibility of developing similar peptide-based assays for the identification of asymptomatic leprosy [6]. Multiple *M*. *leprae*-specific antigens and host markers, including IFN-γ as a candidate host marker, have been studied widely [8–17].

Several studies have explored the immune response in *M*. *leprae*-stimulated WBAs [8–17]. Some of these studies have examined the supernatant levels of IFN-γ stimulated with multiple *M*. *leprae* antigens in infected patients [8–14], and others focused on a panel of multiple *M*. *leprae* antigen-induced host markers by WBA [15–17]. Sampaio et al. [13] reported previously that 9 of 33 *M*. *leprae* recombinant proteins could induce IFN-γ secretion in tuberculoid (TT)/borderline tuberculoid (BT) patients and HHCs by a WBA in a Brazilian population. However, our laboratory recently reported that IFN-γ secretion induced by stimulation with *M*. *leprae* antigens (LID-1, ML89, ML2044, and ML2028) achieved higher positive response rates in PB patients than in MB patients in Southwest China [14,15]. This marker could distinguish PB patients from tuberculosis (TB) patients after stimulation with ML2044 and ML2028, but it could not distinguish PB patients from healthy household contacts (HHC) or endemic controls (ECs) [15]. This result was consistent with those of a previous study in an Ethiopian population, in which *M*. *leprae* proteins did not distinguish patients from ECs in one leprosy endemic area based on IFN-γ [12]. Geluk et al. [12] reported that *M*. *leprae* and ML2478 induced significantly higher concentrations of MCP-1, MIP-1β and IL-1β in PB patients than in ECs in Ethiopia. These studies suggested that *M*. *leprae* antigen-specific IFN-γ secretion in a WBA had a limited ability to discriminate PB patients from nonleprosy controls; indeed, additional *M*. *leprae*-specific antigens and additional host biomarkers should be investigated for their ability to assist in the diagnosis and discrimination of PB patients.

The purpose of this study was to explore a new panel of host markers stimulated by specific *M*. *leprae* antigens in an overnight WBA to improve the diagnosis (PB patients *vs*. ECs) and discrimination (PB patients *vs*. HHCs or TB patients) of PB patients with leprosy in a hyperendemic area in China. The *M*. *leprae* antigens used for the WBA in this study were Leprosy IDRI diagnostic-1 (LID-1) and ML2044. Additional host markers, including tumor necrosis factor alpha (TNF-α), interleukin (IL)-4, interleukin 6, interleukin 10, CC chemokine ligand (CCL) 2, CCL4, CXC chemokine ligand (CXCL) 8, CXCL10, granulocyte colony-stimulating factor (G-CSF) and granulocyte-macrophage colony-stimulating factor (GM-CSF), were also studied.

## Methods

### Ethics statement

This study was approved by the Medical Ethics Committee of Beijing Friendship Hospital, Capital Medical University, Beijing, P.R. China. Written informed consent was obtained from all adult participants. All of the procedures in this study that involved human participants were performed in accordance with the ethical standards of the institutional and/or national research committee and with the 1964 Helsinki Declaration and its later amendments or comparable ethical standards.

### Study subjects

We recruited 13 MB patients, 7 PB patients, 21 HHCs, 20 ECs, and 19 TB patients from the Honghe Autonomous Prefecture in the Yunnan Province in the southwestern region of China during May 2016. The leprosy incidence in most provinces and regions of China was lower than 0.1 per 100,000 in 2010, and the case notification rate was up to 0.85 per 100,000 (190/4,470,000) from 2010–2014 in the communities in the Honghe Autonomous Prefecture [18]. The most common route of identification for the leprosy patients was out-patient (61/190), followed by clue investigation (53/190), family members (40/190), disease-reporting (13/190), epidemic area (12/190), self-reporting (10/190), and group survey (1/190). Leprosy diagnosis was established based on clinical signs and symptoms, skin smears, skin biopsy, and neurophysiologic examinations. The leprosy patients were classified into groups based on Ridley and Jopling [19]. The leprosy patients were also classified into two groups, PB and MB, according to the WHO operational classification [20]. HHCs had been living in the same house as an adult leprosy patient. ECs within the normal controls lived in the same community as leprosy patients. TB patients were referred to the Honghe Autonomous Prefecture Disease Prevention and Control Center (CDC).

### Specific *M*. *leprae* antigens

The specific *M*. *leprae* antigens used for the WBA in this study were LID-1, a fusion protein developed by fusing the ML0405 and ML2331 genes [21,22], and ML2044, which were provided by Dr. M.S. Duthie from the Infectious Disease Research Institute (IDRI) in Seattle, USA. The list of accession numbers/ID numbers for genes and proteins that were mentioned in the text and included in the NCBI search is shown in S1 Table.

### Overnight WBA

An undiluted WBA with overnight incubation was performed as previously described [23]. Briefly, undiluted venous whole blood was collected into a heparinized tube or green top BD vacutainer. A 48-well (flat-bottom) plate was set up with the antigens and controls in a final volume of 0.5 ml. The specific *M*. *leprae* antigens ML2044 and LID-1 (100 μg/ml)were used as stimuli. Phytohemagglutinin (PHA-M, Cat No: L2646, 750 μg/ml, Sigma-aldrich, Fluke, USA) was included as a positive control, and 0.01 mol/l PBS was included as a negative control. The antigens were added in a volume of 50 μl per well, followed by the addition of 450 μl of blood. The plate was sealed with micropore tape to avoid evaporation during incubation at 37°C with 5% CO_2_. After 24 hours of incubation, the supernatants were harvested and stored at −20°C until they were assayed for cytokines and chemokines by Luminex multiplex assays.

### Luminex multiplex assays

The potential diagnostic value of host markers was analyzed with Luminex multiplex assays of supernatant samples collected from an overnight WBA; these markers included TNF-α, IL-4, IL-6, IL-10, CCL 2, CCL4, CXCL 8, CXCL10, G-CSF and GM-CSF. The concentrations of these cytokines were measured by a customized Human Premixed Multi-Analyte Kit (Cat. No. LXSAHM-10) on the Luminex-200™ system and the Xmap Platform (Luminex Corporation, Austin, TX). The list of accession numbers/ID numbers for genes and proteins of the host markers mentioned in the text and included in the HGNC and NCBI search is shown in S2 Table.

### Statistical analysis

Statistical analysis was performed primarily with GraphPad Prism software version 5.0 (GraphPad Software Inc., San Diego, CA, USA). The nonparametric Mann-Whitney U test was used to analyze differences between two groups (PB patients *vs*. MB patients, HHCs, ECs, or TB patients). Probability (p) values less than 0.05 were considered significant. The diagnostic utility of individual *M*. *leprae* antigen-specific responses for leprosy, including sensitivity, specificity, p value, 95% confidence intervals (CI), cutoff value, area under the receiver operator characteristic curve (AUC), and receiver operator characteristics (ROC), was ascertained by ROC curve analysis based on the highest likelihood ratio.

## Results

### Study participants

A total of eighty participants, including leprosy patients (13 MB patients and 7 PB patients) and controls (21 HHCs, 20 ECs, and 19 TB patients) were included in the study. The basic characteristics of the participants are presented in Table 1. Both specific *M*. *leprae* antigens (ML2044 and LID-1) were evaluated in all 13 MB cases, 7 PB cases, 21 HHCs, 19 TB cases, and in different numbers of ECs (19 ECs for ML2044 and 20 ECs for LID-1). Newly diagnosed MB and PB leprosy patients undergoing MDT treatment. The median and interquartile range (IQR) of the treatment duration were 9 months (5–16 months) and 10 months (2–12 months) for MB and PB patients, respectively.

**Table 1:** Clinical characteristics of the leprosy patients enrolled in this study.

	Classification (n, %)		Number of cases	Gender ratio	Median & IQR of MDT	Bacterial index
	WHO*	RJ**	(n, %)	(M/F)	(months)	(BI)
Leprosy	MB	LL	2	2/0	9(5–16)	4–5.3
		BL	11	6/5		0.8–4.3
	PB	BT	7	2/5	10(2–12)	0
Controls	HHC		21	10/11	/	/
	EC		20	10/10	/	/
	TB		19	10/9	/	/

### Host immune markers induced by ML2044 and LID-1 in the overnight WBA

After stimulation with ML2044 (S1 Fig) and LID-1 (S2 Fig) in an overnight WBA, the concentrations of selected host markers were determined in each participant and compared between PB patients and MB patients, HCCs, ECs, and TB patients (Table 2).

**Table 2:** The median and IQR values of host markers detected in overnight culture supernatants from a WBA in leprosy patients (MB and PB patients) and non-leprosy controls (HHCs, ECs, and TB patients).

*M*. *leprae* antigens	Host marker	Median (IQR), pg/ml					P value (Mann-Whitney test)			
		MB	PB	HHC	EC	TB	PB *vs*. MB	PB *vs*. HHC	PB *vs*. EC	PB *vs*. TB
ML2044	TNF-alpha	8.84(2.96–12.74)	8.43(2.84–20.10)	2.96(2.90–2.96)	2.96(2.84–2.96)	2.96(0.83–5.53)	0.78	0.10	0.08	0.04*
	IL-4	46.00(21.91–62.79)	46.00(46.00–62.79)	5.24(5.24–21.91)	5.24(5.24–21.91)	5.24(5.24–21.91)	0.46	<0.01*	<0.01*	<0.01*
	IL-6	62.08(24.47–132.60)	30.66(15.51–123.80)	2.08(1.59–7.45)	3.66(1.50–6.71)	3.27(1.50–5.57)	0.34	0.02*	0.01*	0.01*
	IL-10	1.20(1.06–1.20)	1.20(0.24–1.20)	1.20(1.20–1.20)	1.20(1.20–1.20)	1.20(1.20–1.20)	0.22	/	<0.01*	0.22
	CCL2/MCP-1	232.1(172.3–523.4)	202.2(147–688.4)	172.4(125–315.5)	198.8(109.2–253.2)	233.6(110.5–308.5)	0.93	0.26	0.48	0.41
	CCL4/MIP-1 beta	3328(1471–4923)	2414(1278–4291)	470.9(221.8–759.6)	470.9(374.6–827.8)	193.9(118.7–337.4)	0.52	0.01*	<0.01*	<0.01*
	CXCL8/IL-8	1060(991.3–1060)	1060(1060–2040)	708.5(346.3–1060)	766.1(553.1–1060)	222.7(144.8–553.7)	0.16	0.01*	0.01*	<0.01*
	CXCL10/IP-10	127.2(69.09–187)	76.06(64.72–130.5)	59.5(43.62–96.13)	179.4(102–302.6)	156.5(84.9–237.3)	0.34	0.04*	<0.01*	0.08
	G-CSF	61.9(24.7–185.8)	77.56(56.38–180.5)	17.33(7.572–35.33)	17.33(8.16–32.06)	17.33(7.57–56.38)	0.36	<0.01*	<0.01*	<0.01*
	GM-CSF	1.65(0.14–3.97)	3.97(3.97–3.97)	3.97(3.97–3.97)	3.97(0.93–3.97)	3.97(1.27–3.97)	0.14	0.72	0.61	0.41
LID-1	TNF-alpha	4.77(2.96–11.09)	2.96(2.31–3.81)	2.96(1.78–2.96)	2.96(2.04–3.60)	2.96(1.78–2.96)	0.10	0.39	0.97	0.49
	IL-4	24.54(8.79–35.10)	12.35(5.24–24.54)	5.24(5.24–12.35)	5.24(5.24–12.35)	5.24(5.24–12.35)	0.27	0.28	0.11	0.13
	IL-6	29.07(12.09–109.10)	21.95(4.10–45.82)	4.742(1.70–15.43)	5.79(2.34–13.97)	4.528(2.34–11.90)	0.15	0.06	0.15	0.09
	IL-10	1.20(0.68–1.20)	1.20(1.20–1.20)	1.20(1.20–1.20)	1.20(1.09–1.20)	1.20(1.20–1.20)	0.62	0.47	0.71	0.89
	CCL2/MCP-1	95.12(78.94–187.10)	97.45(79.79–179.30)	122.2(96.23–175.90)	90.25(62.64–145.80)	87.77(57.21–195.70)	0.93	0.45	0.48	0.62
	CCL4/MIP-1 beta	1472(804.5–2914)	1379(505.8–1697)	495.4(316.2–923.6)	338(168.7–540.8)	362.2(89.8–683.1)	0.30	0.08	0.02*	0.02*
	CXCL8/IL-8	1343(1006–1592)	1325(429.4–1975)	697.5(422.2–2333)	349.8(155.1–972.7)	520.6(252.6–776.4)	0.93	0.71	0.03*	0.09
	CXCL10/IP-10	88.9(59.7–153.2)	62.32(44.4–83.9)	54.8(40.5–93.5)	93.97(50.2–199.7)	124.3(82.8–374.4)	0.17	0.52	0.21	0.01*
	G-CSF	44.15(31.62–113.20)	70.75(58.22–82.31)	51.43(27.07–58.22)	44.15(29.35–67.62)	58.22(27.07–82.31)	0.45	0.02*	0.07	0.23
	GM-CSF	3.97(3.97–3.97)	3.97(3.97–3.97)	3.97(3.97–3.97)	3.97(3.97–3.97)	3.97(3.97–3.97)	/	/	/	/

Whole blood was collected from all participates and stimulated overnight with specific *M*. *leprae* antigens (ML2044 and LID-1). The concentrations of cytokines and chemokines were determined with Luminex multiplex assays.

After stimulation with ML2044, the supernatants of PB patients had a higher level of IL-4 (median 46 pg/ml) than those of HHCs (median 5.24 pg/ml, p = 0.0004), ECs (median 5.24 pg/ml, p = 0.0026), or TB patients (median 5.24 pg/ml, p = 0.0005). The same trend was also found for IL-6 (PB patients *vs*. HHCs, ECs, and TB patients: median = 30.66 *vs*. 2.086, 3.661, and 3.271 pg/ml, p = 0.0225, 0.0109, and 0.0192, respectively); CCL4/MIP-1 beta (PB patients *vs*. HHCs, ECs, and TB patients: median = 2414 *vs*. 470.9, 470.9, and 193.9 pg/ml, p = 0.0101, 0.0066, and 0.0022, respectively); CXCL8/IL-8 (PB patients *vs*. HHCs, ECs, and TB patients: median = 1060 *vs*. 708.5, 766.1, and 222.7 pg/ml, p = 0.0139, 0.0185, and 0.0019, respectively); G-CSF (PB patients *vs*. HHCs, ECs, and TB patients: median = 77.56 *vs*. 17.33, 17.33, and 17.33 pg/ml, p = 0.0013, 0.0006, 0.0046, respectively); and TNF-α (PB patients *vs*. TB patients: median = 8.438 *vs*. 2.963 pg/ml, p = 0.0423). For ML2044-stimulated CXCL10/IP-10, the concentration detected in PB patients (median = 76.06 pg/ml) was higher than that detected in HHCs (median = 59.5 pg/ml, p = 0.0496) but lower than that detected in ECs (median = 179.4, p = 0.0093). Although the median of the ML2044-induced IL-10 concentration differed significantly between the PB and EC groups (median 1.207 *vs*. 1.207, IQR 0.2437–1.207 *vs*. 1.207–1.207, p = 0.0058), the ROC analysis precluded the determination of a discriminatory value for PB patients and ECs.

The concentration of CCL4/MIP-1 beta in whole blood cells during the response to LID-1 was elevated in PB patients compared with that in ECs or TB patients (median 1379 *vs*. 338, and 362.2 pg/ml, p = 0.0217, and 0.0242, respectively), as was the concentration of CXCL8/IL-8 between PB patients and ECs (median 1325 *vs*. 349.8 pg/ml, p = 0.0355) and the concentration of G-CSF between PB patients and HHCs (median 70.75 *vs*. 51.43 pg/ml, p = 0.0214). In contrast, the concentration of LID-1-induced CXCL10/IP-10 was lower in PB patients than in TB patients (median 62.32 *vs*. 124.3 pg/ml, respectively, p = 0.0129).

No differences in host immune markers induced by ML2044 or LID-1 stimulation were found between PB patients and MB patients.

### Potential value of host markers induced by ML2044 and LID-1 in a WBA for the diagnosis of PB patients

**Fig 1:**
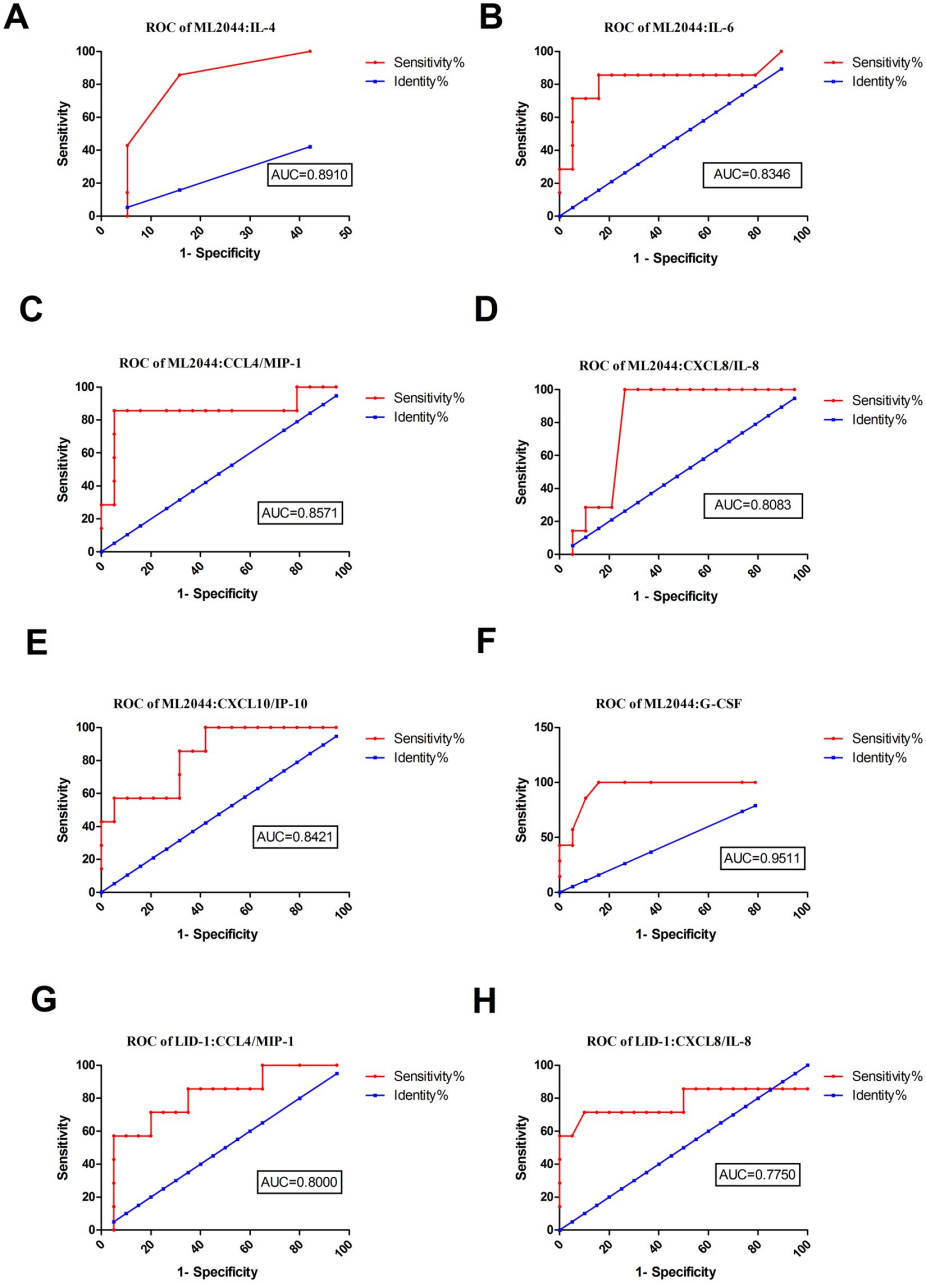
ROC curves showing the accuracy of host markers in discriminating between PB patients and ECs. ROC curves for the accuracy of single markers [IL-4 (A), IL-6 (B), CCL4 (C), CXCL8 (D), CXCL10 (E), and G-CSF (F) induced by ML2044 and CCL4 (G) and CXCL-8 (H) induced by LID-1] in differentiating between PB patients and ECs. Only ROC curves for markers that differentiated between the two infection states with p value≤0.05 and AUC ≥ 0.73 are shown.

The levels of six out of the 10 markers evaluated in this study (IL-4, IL-6, CCL4/MIP-1 beta, CXCL8/IL-8, CXCL10/IP-10, and G-CSF) were significantly higher in ML2044-stimulated supernatants from PB patients than in those from ECs (S3 Table). The AUC values ranged from 0.81 to 0.95 (S3 Table, Fig 1A–1F). For LID-1, the levels of two markers (CCL4/MIP-1 beta and CXCL8/IL-8) were significantly higher in PB patients than in ECs. These two analytes discriminated between PB patients and ECs with AUC = 0.80, and 0.77, respectively, with a sensitivity of 57.14% and a specificity of 95.00% for both markers (S3 Table, Fig 1G and 1H). Combining the results of the host markers stimulated by the two *M*. *leprae* antigens, ML2044-stimulated CXCL8/IL-8 achieved the highest sensitivity of 100%, with a specificity of 73.68%, for PB diagnosis.

### Potential value of host markers induced by ML2044 and LID-1 in a WBA for discriminating PB patients from HHCs

**Fig 2:**
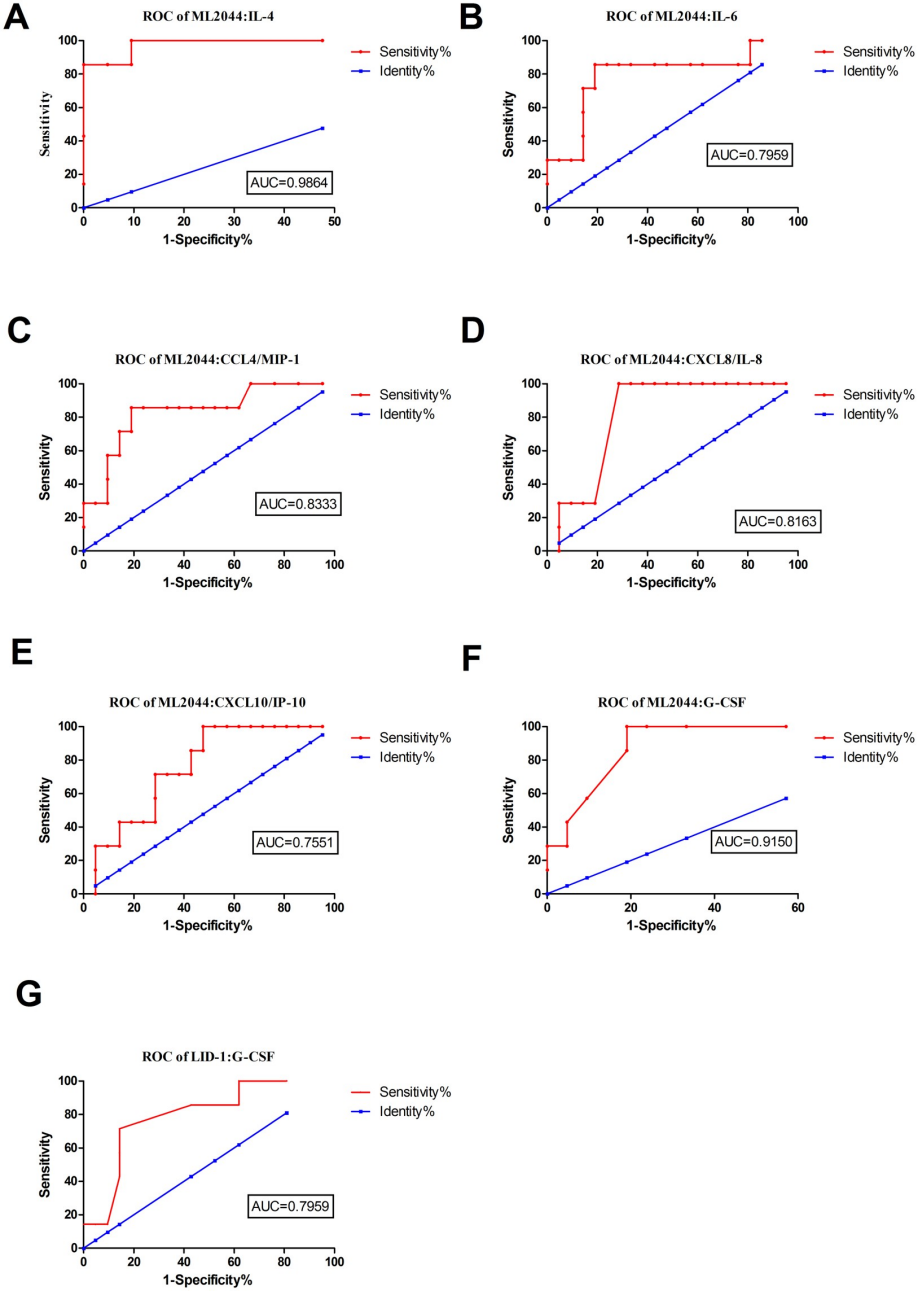
ROC curves showing the accuracy of host markers in discriminating between PB patients and HHCs. ROC curves for the accuracy of single markers [IL-4 (A), IL-6 (B), CCL4 (C), CXCL8 (D), CXCL10 (E), and G-CSF (F) induced by ML2044 and G-CSF (G) induced by LID-1] for differentiating between PB patients and HHCs. Only ROC curves for markers that differentiated between the two infection states with p value≤0.05 and AUC ≥ 0.73 are shown.

When ML2044-stimulated analyte levels were compared between the PB patients and HHCs, similar to the results obtained for PB patients *vs*. ECs, significant differences were obtained for IL-4, IL-6, CCL4/MIP-1 beta, CXCL8, CXCL10/IP-10, and G-CSF. The AUC values for all six of these markers were ≥0.73 by ROC analysis. The sensitivities of the six analytes for PB patients ranged from 28.75% to 85.71%, with specificities from 90.48% to 95.24% (Fig 2, S4 Table). For LID-1-stimulated supernatants, significant differences between the PB patients and HHCs were obtained for one host marker, G-CSF. After ROC analysis, the AUC for LID-1 stimulated G-CSF was 0.80. Among the host markers stimulated by the two *M*. *leprae* antigens, ML2044-stimulated IL-4 achieved the highest sensitivity (85.71%) and specificity (95.24%) for discriminating PB patients from HHCs.

### Potential value of host markers induced by ML2044 and LID-1 in a WBA for discriminating PB patients from TB patients

**Fig 3:**
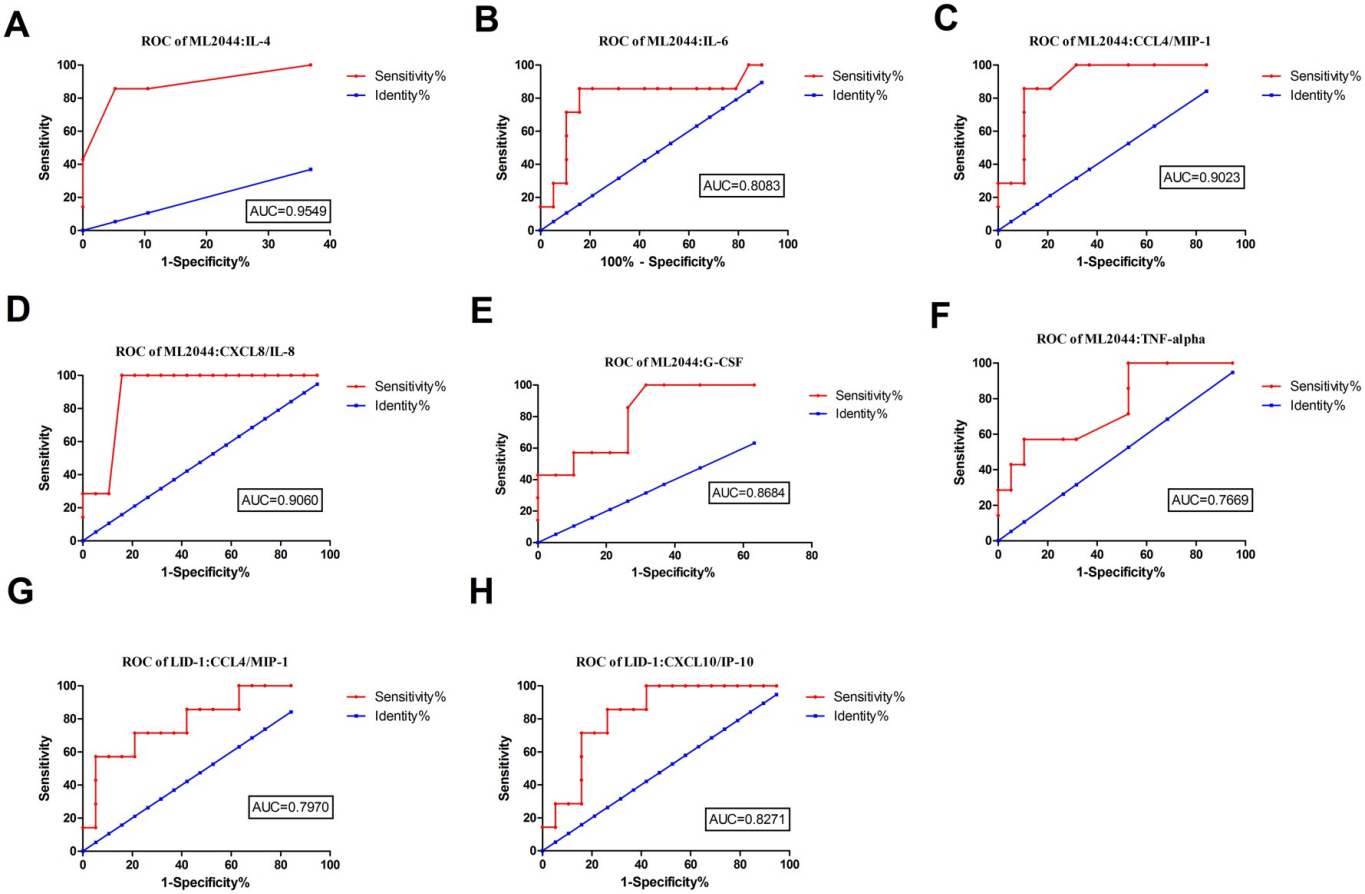
ROC curves showing the accuracy of host markers in discriminating between PB and TB patients. ROC curves for the accuracy of single markers [IL-4 (A), IL-6 (B), CCL4 (C), CXCL8 (D), G-CSF (E), and TNF-α (F) induced by ML2044 and CCL4 (G) and CXCL10 (H) induced by LID-1] to differentiate between PB patients and TB patients. Only ROC curves for markers that differentiated between the two infection states with p value≤0.05 and AUC ≥ 0.73 are shown.

ML2044-induced IL-4, IL-6, CCL4/MIP-1 beta, CXCL8/IL-8, G-CSF and TNF-α levels were significantly higher in the PB patients than in the TB patients. After ROC analysis, the AUC values for all six analytes ranged from 0.7669 to 0.9549 (Fig 3, S5 Table). For LID-1-stimulated supernatants, significant differences between the PB patients and TB patients were obtained for two host markers, CCL4/MIP-1 beta and CXCL10/IP-10. After ROC analysis, the AUC values for these markers were 0.80 and 0.83, respectively. Among the host markers stimulated by the two *M*. *leprae* antigens, ML2044-stimulated CXCL8/IL-8 achieved the highest sensitivity of 100%, with a specificity of 84.21%, for discriminating PB patients from TB patients.

### Diagnostic value of combination models for PB patients

**Fig 4:**
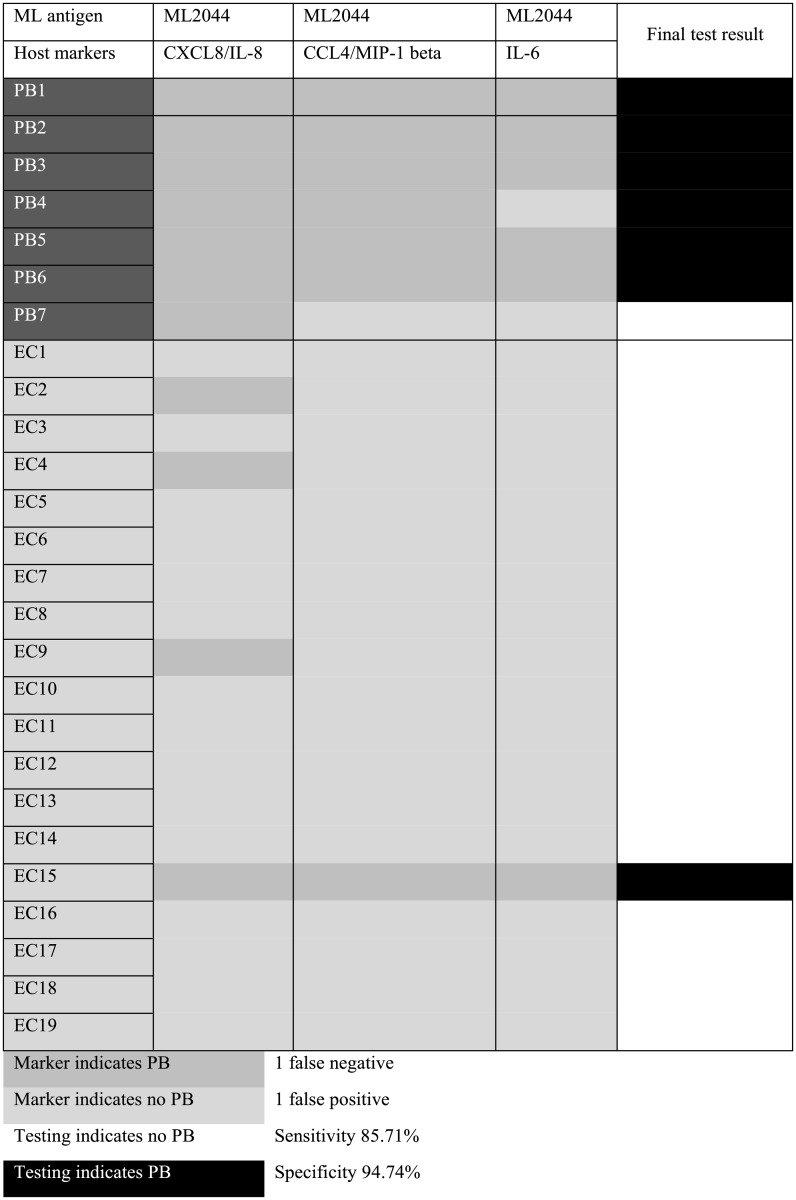
Utility of a 3-host marker combination model for the diagnosis of paucibacillary (PB) leprosy patients.

Although the sensitivity of ML2044-induced CXCL8/IL-8 (up to 100% for the discrimination of PB patients from ECs) predominated over any other single host marker induced by ML2044 or LID-1, the specificity of this marker reached only 73.68%, far lower than those of IL-6, CXCL10/IP-10, CCL4/MIP-1 beta, IL-4, and G-CSF induced by ML2044 (specificity = 94.74% for each marker) or CXCL8/IL-8 and CCL4/MIP-1 beta induced by LID-1 (specificity = 95% for each marker), as shown in S3 Table. To increase the specificity of diagnosis between PB patients and ECs, different combination models of 3–6 *M*. *leprae*-specific antigen-induced host markers were used to classify the participants. The cutoff value obtained using the ROC analysis described above was used to classify individuals into two groups. A 3-marker model (ML2044-induced CXCL8/IL-8, CCL4/MIP-1 beta, and IL-6) was the best model and was superior to the use of two *M*. *leprae*-specific antigens (ML2044 and LID-1) or 4, 5 or 6 host markers in combination. Participants were allocated to clinical groups according to the results of the majority of the individual marker tests (2/3 or 3/3). Accordingly, the ML2044-induced 3-host marker combination model classified 24 of the 26 participants (92.31%) in the correct clinical groups, with 85.71% sensitivity, which was lower than the sensitivity of ML2044-induced IL-8 as a single marker (100%), but the specificity of diagnosis for PB leprosy patients increased from 73.68% to 94.74%. Two (7.7%) participants were misclassified, with 1 false-negative and 1 false-positive classification. The classification of individual participants, sensitivity values and specificity values are shown in Fig 4. Despite the high cost and low efficiency, combined utilization of ML2044-induced CXCL8/IL-8 as a single marker and the 3-marker model (ML2044-induced CXCL8/IL-8, CCL4/MIP-1 beta, and IL-6) would help to both maintain high sensitivity and enhance specificity.

Seven PB patients, as defined by Jopling, and 19 HCCs were analyzed. The concentrations and the best cutoff were determined for each cytokine or chemokine, as described in S3 Table. The cutoff was used to define whether the concentrations of cytokines and chemokines indicated that the participant was a PB patient or an EC. Each cytokine or chemokine was used as an independent marker. Prediction of PB patients is shown in dark gray, and prediction of ECs is shown in light gray, for 3 different phenotypic markers. When ≥ 2 phenotypes supported one of the diagnoses, a final diagnosis of either PB (black) or EC (white) was made.

Subsequently, we explored a model with 3 or more markers for distinguishing PB patients from HHCs or TB patients in the same way. The sensitivity and the specificity of a single marker (ML2044-induced IL-4) reached 85.71% and 100% for distinguishing PB patients from HHCs. Only the 3-marker model (ML2044-induced IL-4, LID-1-induced G-CSF, and ML2044-induced CCL4/MIP-1 beta) achieved the same sensitivity and specificity as the single marker (ML2044-induced IL-4), as shown in S3 Fig.

In addition, the sensitivity and the specificity of a single marker (ML2044-induced CXCL8) reached 100% and 94.74%. Only the 3-marker model (ML2044-induced CXCL8, CCL4/MIP-1 beta, and IL-4) was found to have the same sensitivity and specificity as ML2044-induced CXCL8, as shown in S4 Fig.

## Discussion

The use of the *M*. *leprae* antigens ML2044 and LID-1 to induce host immune responses in a WBA for the diagnosis of PB patients with leprosy and discrimination between PB patients and MB patients, HHCs, or TB patients were evaluated in this study. The key findings are presented below. (1) ML2044-stimulated CXCL8/IL-8 reached the highest sensitivity of 100%, with a specificity of 73.68%, for PB diagnosis. A 3-marker combination model that included ML2044-induced CXCL8/IL-8, CCL4/MIP-1 beta, and IL-6 improved the diagnostic specificity to 94.7% in comparison to that of the single marker. (2) ML2044- stimulated IL-4 and ML2044-stimulated CXCL8/IL-8 reached the highest sensitivity (85.71% and 100%, respectively) and the highest specificity (95.24% and 84.21%, respectively) for discriminating PB patients from HHCs and TB patients, respectively. No combination model with 3 or more markers had more promising diagnostic utility than analysis of this single marker for discriminating PB patients from HHCs and TB patients. (3) Although no selected host markers showed potential value for discriminating PB patients from MB patients, MB patients and PB patients showed similar but not identical *M*. *leprae*-induced immune responses by WBA.

None of the single markers could correctly classify all participants into their respective groups; however, ML2044-stimulated CXCL8/IL-8 reached the highest sensitivity of 100%, with a moderate specificity of 73.68%, for PB diagnosis. Indeed, the 3-marker model (ML2044-induced CXCL8/IL-8, CCL4/MIP-1 beta, and IL-6) enhanced the specificity to 94.74%. As ML2044 induced more host markers and had higher sensitivity than LID-1, immune responses induced by the *M*. *leprae*-specific antigen ML2044 were superior to LID-1-induced immune responses for the diagnosis and discrimination of PB patients from ECs, HHCs, and TB patients.

It was reported that at diagnosis, PB patients produce IFN-γ, and MB patients exhibit a weak/absent response. Shortly after MDT, IFN-γ production in PB patients decreases, except in response to LID-1; MB patients produced IFN-γ in response to LID-1. Almost 2 years after MDT, IFN-γ levels decreased in PB and MB patients [9]. This finding implied that cytokines and chemokines, such as IFN-γ, may fluctuate during immune responses to *M*. *leprae* antigens. In this study, the median and IQR of the treatment duration were 9 months (5–16 months) and 10 months (2–12 months) for enrolled MB patients and PB patients, respectively. It remained unknown whether the kinetics of the response of other host immune markers to *M*. *leprae* antigens during MDT treatment also fluctuated.

Interleukin 8 (CXCL8) is a chemoattractant and a regulator of white blood cell production, which can affect the pathogenesis of intense infectious diseases, such as TB, by suppressing the normal immune response to *M*. *tuberculosis* that can lead to granuloma formation. Although monocytes and macrophages infected with *M*. *tuberculosis* are the main sources of CXCL8 production, this chemokine can also be produced by neutrophils and respiratory epithelial cells [24]. However, the relationship between *M*. *leprae* and CXCL8 remained unclear. IL-8 may assume a pivotal role in cell recruitment in leprosy patients with disseminated mycobacterial infections [25]. The presence of the neutrophil chemoattractant IL-8 in leprosy lesions, which do not contain neutrophils, strongly suggests a role of IL-8 in monocyte and lymphocyte recruitment in leprosy lesions [26]. TNF-induced IL-8 can be produced by Schwann cells (SCs), indicating involvement of this factor in leprosy-associated nerve damage [27].

A previous study showed that CXCL10 could discriminate PB patients from ECs in an ML0276 + LID-1 WBA and did not discriminate active disease (PB) from *M*. *leprae*-infected (HHC) individuals in Brazil [8]. Another study also focused on *M*. *leprae* antigens (ML0276, ML1623, ML0405, ML1632, 92f, and ML1011) that stimulated host markers (eotaxin, IFN-γ, IL-2, IL-4, IL-5, IL-6, IL-10, IL-12p70, IL-15, IL-17A, IL-23, IL-31, IP-10, and TNF-α) in a WBA in a Brazil population, which demonstrated that IFN-γ is currently the best indicator of an antigen-specific cellular immune response and that none of the biomarkers tested could discriminate leprosy patients from HHCs [17]. In this study, CXCL10 could discriminate not only PB patients from ECs in an ML2044 WBA but also PB patients from HHCs in an ML2044 WBA and PB patients from TB patients in a LID-1 WBA. Heterogeneity among individuals, different *M*. *leprae* antigen characteristics, and different time points of sample collection may represent influencing factors.

The present study has some limitations. The number of PB patients was rather low, and the findings should be interpreted with caution. Further investigation in larger populations will give more confidence to the diagnostic and discriminatory value of the identified host markers, such as ML-2044-induced IL-8. We also lack data on cytokine concentrations in biopsies of skin lesions. Moreover, associations do not necessarily indicate causal relationships, and further mechanistic studies are needed to elucidate the role of the immune response during *M*. *leprae* infection.

### Conclusion

In conclusion, we identified a biosignature of a single *M*. *leprae*-specific host marker in antigen-stimulated overnight WBAs (ML2044-induced CXCL8/IL-8) that showed potential for the diagnosis of PB disease, with accurate prediction of 100% of PB cases and 73.68% of EC cases. The sensitivity of this analyte model was better than that of any other single host marker, but the specificity was relatively low. A 3-marker model of ML2044-induced CXCL8/IL-8, CCL4/MIP-1 beta, and IL-6 improved the specificity of diagnosis between PB patients and ECs to 94.74%. Moreover, ML2044-induced CXCL8/IL-8 and ML2044-induced IL-4 dominated in discriminating PB patients from TB patients and HHCs, respectively. However, both diagnostic performance at the time of screening and assessment of immunological changes in host markers during MDT therapy need to be further evaluated before these diagnostic approaches can be recommended for routine clinical practice.

## Supporting information

S1 Table List of accession numbers/ID numbers for genes and proteins of *M*. *leprae* antigens that were mentioned in the text and included in the NCBI search. (DOCX)

S2 Table List of accession numbers/ID numbers for genes and proteins of host markers that were mentioned in the text and included in the HGNC and NCBI searches. (DOCX)

S3 Table Diagnostic potential of host markers detected in culture supernatants from an overnight WBA for discriminating paucibacillary (PB) leprosy patients from endemic controls (ECs). (DOCX)

S4 Table Diagnostic potential of host markers detected in culture supernatants from an overnight WBA for discriminating paucibacillary (PB) leprosy patients from HHCs. (DOCX)

S5 Table Diagnostic potential of host markers detected in culture supernatants from an overnight WBA for discriminating paucibacillary (PB) leprosy patients from tuberculosis (TB) patients. (DOCX)

S1 Fig Levels of individual analytes in leprosy cases and non-leprosy controls stimulated with ML2044 by WBA. Each dot represents the analyte level of one participant in the study, and horizontal lines represent the median and IQR values. The cytokine and chemokine levels [TNF-α (A), IL-4 (B), IL-6 (C), IL-10 (D), CCL2 (E), CCL4 (F), CXCL8 (G), CXCL10 (H), G-CSF (I) and GM-CSF (J)] obtained in the supernatant after overnight stimulation with the specific *M*. *leprae* antigen ML2044 by WBA.
(TIF)

S2 Fig Levels of individual analytes in leprosy cases and non-leprosy controls stimulated with LID-1 by WBA. Each dot represents the analyte level of one participant in the study, and horizontal lines represent the median and IQR values. The cytokine and chemokine levels [TNF-α (A), IL-4 (B), IL-6 (C), IL-10 (D), CCL2 (E), CCL4 (F), CXCL8 (G), CXCL10 (H), G-CSF (I) and GM-CSF (J)] obtained in supernatants after overnight stimulation with the specific *M*. *leprae* antigen LID-1 by WBA.
(TIF)

S3 Fig Utility of a 3-host marker combination model for distinguishing the diagnosis of PB from HHCs. Seven PB patients, as defined by the WHO, and 21 HCCs were analyzed. The concentration and the best cutoff were determined for each cytokine or chemokine, as described in S4 Table. The cutoff was used to define whether the concentrations of cytokines and chemokines indicated that the participant was a PB patient or an HHC. Each cytokine or chemokine was used as an independent marker. Prediction of PB patients is shown in dark gray, and prediction of HHCs is shown in light gray for 3 different phenotypic markers. When ≥ 2 phenotypes supported one of the diagnoses, a final diagnosis of either PB (black) or HHC (white) was made.
(PDF)

S4 Fig Utility of a 3-host marker combination model for distinguishing the diagnosis of PB from TB. Seven PB patients, as defined by the WHO, and 21 HCCs were analyzed. The concentration and the best cutoff were determined for each cytokine or chemokine, as described in S5 Table. The cutoff was used to define whether the concentrations of cytokines and chemokines indicated that the participants was a PB patient or a TB patient. Each cytokine or chemokine was used as an independent marker. Prediction of PB patients is shown in dark gray, and prediction of TB patients is shown in light gray for 3 different phenotypic markers. When ≥ 2 phenotypes supported one of the diagnoses, a final diagnosis of either PB (black) or TB (white) was made.
(PDF)
