# Supplementary material for: Host immune responses induced by specific Mycobacterium leprae antigens in an overnight whole-blood assay correlate with the diagnosis of paucibacillary leprosy patients in China
Source: PLoS Negl Trop Dis. 2019 Apr 24;13(4):e0007318. doi: 10.1371/journal.pntd.0007318 (PMC6481774; doi:10.1371/journal.pntd.0007318)
Supplement: S1 Table — (DOCX) [file pntd.0007318.s001.docx]

**S1 Table. List of Accession Numbers/ID Numbers for Genes and Proteins of *M. leprae* Antigens that were Mentioned in the Text and Included in the NCBI Search.**

| Name | Gene ID | Description | Location | Aliases |
| --- | --- | --- | --- | --- |
| ML0405 | ID: 909138 | hypothetical protein [*Mycobacterium leprae TN*] | NC_002677.1 (503217..504401) | ML0405 |
| ML2331 | ID: 908688 | hypothetical protein [*Mycobacterium leprae TN*] | NC_002677.1 (2761703..2762473) | ML2331 |
| ML2044 | ID: 909000 | hypothetical protein [*Mycobacterium leprae TN*] | NC_002677.1 (2434368..2434589, complement) | ML2044 |
