# Supplementary material for: Host immune responses induced by specific Mycobacterium leprae antigens in an overnight whole-blood assay correlate with the diagnosis of paucibacillary leprosy patients in China
Source: PLoS Negl Trop Dis. 2019 Apr 24;13(4):e0007318. doi: 10.1371/journal.pntd.0007318 (PMC6481774; doi:10.1371/journal.pntd.0007318)
Supplement: S2 Table — (DOCX) [file pntd.0007318.s002.docx]

**S2 Table. List of Accession Numbers/ID Numbers for Genes and Proteins of Host Markers that were Mentioned in the Text and Included in the HGNC and NCBI Searches**

| **Approved symbol** | TNF | IL4 | IL6 | IL10 | CCL2 | CCL4 | CXCL8 | CXCL10 | CSF3 | CSF2 |
| --- | --- | --- | --- | --- | --- | --- | --- | --- | --- | --- |
| **Approved name** | tumor necrosis factor | interleukin 4 | interleukin 6 | interleukin 10 | C-C motif chemokine ligand 2 | C-C motif chemokine ligand 4 | C-X-C motif chemokine ligand 8 | C-X-C motif chemokine ligand 10 | colony-stimulating factor 3 | colony-stimulating factor 2 |
| **Previous symbols** | TNFA | / | IFNB2 | / | SCYA2 | LAG1; SCYA4 | IL-8 | INP10; SCYB10 | GCSF; G-CSF | / |
| **Previous names** | tumor necrosis factor (TNF superfamily, member 2) | / | interleukin 6 (interferon, beta 2) | / | chemokine (C-C motif) ligand 2 | chemokine (C-C motif) ligand 4 | interleukin 8, chemokine (C-X-C motif) ligand 8 | chemokine (C-X-C motif) ligand 10 | colony-stimulating factor 3 (granulocyte) | colony-stimulating factor 2 (granulocyte-macrophage) |
| **Alias symbols** | TNF-alpha | BSF1; IL-4 | BSF2; IL-6 | IL10A; IL-10 | MCP1; MCP-1 | MIP-1-beta | IL-8 | IP-10 | MGC45931 | GM-CSF; GMCSF |
| **Alias names** | TNF superfamily, member 2 | B cell stimulatory factor 1 | interferon, beta 2 | cytokine synthesis inhibitory factor | monocyte chemoattractant protein-1 | / | neutrophil-activating peptide 1 | / | granulocyte colony-stimulating factor | granulocyte-macrophage colony-stimulating factor |
| **HGNC ID** | HGNC: 11892 | HGNC: 6014 | HGNC: 6018 | HGNC: 5962 | HGNC: 10618 | HGNC: 10630 | HGNC: 6025 | HGNC: 10637 | HGNC: 2438 | HGNC: 2434 |
| **NCBI** | Gene ID: 7124 | Gene ID: 3565 | Gene ID: 3569 | Gene ID: 3586 | Gene ID: 6347 | Gene ID: 6351 | Gene ID: 3576 | Gene ID: 3627 | Gene ID: 1440 | Gene ID: 1437 |
