# Supplementary material for: Host immune responses induced by specific Mycobacterium leprae antigens in an overnight whole-blood assay correlate with the diagnosis of paucibacillary leprosy patients in China
Source: PLoS Negl Trop Dis. 2019 Apr 24;13(4):e0007318. doi: 10.1371/journal.pntd.0007318 (PMC6481774; doi:10.1371/journal.pntd.0007318)
Supplement: S3 Table — (DOCX) [file pntd.0007318.s003.docx]

**S3 Table**

| *M. leprae* antigens | Host marker | PB: median (IQR), | EC: median (IQR), | P value | AUC | 95% CI | Cutoff | Sensitivity | Specificity |
| --- | --- | --- | --- | --- | --- | --- | --- | --- | --- |
|  |  | pg/ml | pg/ml |  |  |  |  | % | % |
| ML2044 | TNF-alpha | 8.43(2.84-20.1) | 2.96(2.84-2.96) | 0.09 | 0.72 | 0.44 to 0.99 | > 3.81 | 57.14% | 94.74% |
|  | IL-4 | 46(46-62.79) | 5.24(5.24-21.91) | <0.01* | 0.89 | 0.76 to 1.02 | > 54.40 | 42.86% | 94.74% |
|  | IL-6 | 30.66(15.51-123.80) | 3.661(1.50-6.71) | 0.01* | 0.83 | 0.61 to 1.05 | > 27.35 | 71.43% | 94.74% |
|  | IL-10 | 1.20(0.240-1.20) | 1.20(1.20-1.20) | 0.07 | 0.73 | 0.48 to 0.97 | < 1.62 | 100.00% | 5.26% |
|  | CCL2/MCP-1 | 202.2(147.0-688.4) | 198.8(109.2-253.2) | 0.47 | 0.59 | 0.34 to 0.83 | > 603.20 | 28.57% | 89.47% |
|  | CCL4/MIP-1 beta | 2414(1278-4291) | 470.9(374.6-827.8) | <0.01* | 0.86 | 0.64 to 1.06 | > 1240 | 85.71% | 94.74% |
|  | CXCL8/IL-8 | 1060(1060-2040) | 766.1(553.1-1060) | 0.01* | 0.81 | 0.64 to 0.97 | > 1037 | 100.00% | 73.68% |
|  | CXCL10/IP-10 | 76.06(64.72-130.5) | 179.4(102.0-302.6) | <0.01* | 0.84 | 0.67 to 1.00 | < 78.97 | 57.14% | 94.74% |
|  | G-CSF | 77.56(56.38-180.5) | 17.33(8.16-32.06) | <0.01* | 0.95 | 0.87 to 1.02 | > 66.97 | 57.14% | 94.74% |
|  | GM-CSF | 3.97(3.97-3.97) | 3.97(0.93-3.97) | 0.66 | 0.56 | 0.31 to 0.79 | > 2.81 | 85.71% | 31.58% |
| LID-1 | TNF-alpha | 2.96(2.31-3.81) | 2.96(2.04-3.60) | 0.97 | 0.50 | 0.26 to 0.74 | < 1.78 | 14.29% | 90.00% |
|  | IL-4 | 12.35(5.24-24.54) | 5.24(5.24-12.35) | 0.15 | 0.68 | 0.41 to 0.94 | > 15.54 | 42.86% | 95.00% |
|  | IL-6 | 21.95(4.1-45.82) | 5.79(2.345-13.97) | 0.14 | 0.69 | 0.45 to 0.92 | > 21.77 | 57.14% | 90.00% |
|  | IL-10 | 1.20(1.20-1.20) | 1.207(1.092-1.207) | 0.76 | 0.54 | 0.30 to 0.77 | > 0.50 | 100.00% | 15.00% |
|  | CCL2/MCP-1 | 97.45(79.79-179.3) | 90.25(62.64-145.80) | 0.47 | 0.59 | 0.36 to 0.82 | > 259.60 | 14.29% | 95.00% |
|  | CCL4/MIP-1 beta | 1379(505.8-1697) | 338(168.7-540.8) | 0.02* | 0.80 | 0.60 to 0.99 | > 11910 | 57.14% | 95.00% |
|  | CXCL8/IL-8 | 1325(429.4-1975) | 349.8(155.1-972.7) | 0.03* | 0.78 | 0.50 to 1.04 | > 11410 | 57.14% | 95.00% |
|  | CXCL10/IP-10 | 62.32(44.43-83.98) | 93.97(50.28-199.7) | 0.20 | 0.66 | 0.45 to 0.86 | < 45.10 | 28.57% | 90.00% |
|  | G-CSF | 70.75(58.22-82.31) | 44.15(29.35-67.62) | 0.07 | 0.73 | 0.52 to 0.93 | > 61.42 | 71.43% | 75.00% |
|  | GM-CSF | 3.97(3.97-3.97) | 3.97(3.97-3.97) | 1.00 | 0.50 | 0.24 to 0.75 | / | / | / |

Whole blood was collected from newly diagnosed PB leprosy patients and ECs and stimulated overnight with *M. leprae*-specific antigens (ML2044 and LID-1). The concentrations of cytokines and chemokines were determined with Luminex multiplex assays. The AUC and 95% confidence intervals (95% CIs) were calculated with ROC analysis. p-values with asterisks indicate significant differences
