# Supplementary material for: Host immune responses induced by specific Mycobacterium leprae antigens in an overnight whole-blood assay correlate with the diagnosis of paucibacillary leprosy patients in China
Source: PLoS Negl Trop Dis. 2019 Apr 24;13(4):e0007318. doi: 10.1371/journal.pntd.0007318 (PMC6481774; doi:10.1371/journal.pntd.0007318)
Supplement: S4 Table — (DOCX) [file pntd.0007318.s004.docx]

**S4 Table**

| *M. leprae* antigens | Host marker | PB: median (IQR), | HHC: median (IQR), | P value | AUC | 95% CI | Cutoff | Sensitivity | Specificity |
| --- | --- | --- | --- | --- | --- | --- | --- | --- | --- |
|  |  | pg/ml | pg/ml |  |  |  |  | % | % |
| ML2044 | TNF-alpha | 8.43(2.84-20.1) | 2.96(2.90-2.96) | 0.11 | 0.70 | 0.43 to 0.97 | > 6.10 | 57.14% | 95.24% |
|  | IL-4 | 46.00(46.00-62.79) | 5.24(5.24-21.91) | <0.01* | 0.99 | 0.95 to 1.02 | > 40.81 | 85.71% | 95.24% |
|  | IL-6 | 30.66(15.51-123.80) | 2.08(1.59-7.45) | 0.02* | 0.80 | 0.58 to 1.01 | > 73.84 | 28.57% | 95.24% |
|  | IL-10 | 1.20(0.24-1.20) | 1.20(1.20-1.20) | 0.09 | 0.71 | 0.45 to 0.97 | / | / | / |
|  | CCL2/MCP-1 | 202.2(147.-688.4) | 172.4(125-315.5) | 0.25 | 0.64 | 0.41 to 0.88 | > 492.80 | 28.57% | 95.24% |
|  | CCL4/MIP-1 beta | 2414(1278-4291) | 470.9(221.8-759.6) | <0.01* | 0.83 | 0.65 to 1.01 | > 2089.00 | 57.14% | 90.48% |
|  | CXCL8/IL-8 | 1060(1060-2040) | 708.5(346.3-1060) | 0.01* | 0.82 | 0.66 to 0.97 | > 16210 | 28.57% | 95.24% |
|  | CXCL10/IP-10 | 76.06(64.72-130.5) | 59.50(43.62-96.13) | 0.04* | 0.76 | 0.57 to 0.93 | > 126.60 | 28.57% | 95.24% |
|  | G-CSF | 77.56(56.38-180.5) | 17.33(7.57-35.33) | <0.01* | 0.92 | 0.81 to 1.01 | > 109.20 | 42.86% | 95.24% |
|  | GM-CSF | 3.97(3.97-3.97) | 3.97(3.97-3.97) | 0.83 | 0.53 | 0.26 to 0.78 | < 1.24 | 14.29% | 95.24% |
| LID-1 | TNF-alpha | 2.96(2.31-3.81) | 2.96(1.78-2.96) | 0.39 | 0.61 | 0.39 to 0.82 | > 3.39 | 28.57% | 80.95% |
|  | IL-4 | 12.35(5.24-24.54) | 5.24(5.24-12.35) | 0.30 | 0.63 | 0.37 to 0.88 | > 21.64 | 42.86% | 90.48% |
|  | IL-6 | 21.95(4.1-45.82) | 4.742(1.7-15.43) | 0.05 | 0.74 | 0.51 to 0.96 | > 24.03 | 42.86% | 95.24% |
|  | IL-10 | 1.20(1.20-1.20) | 1.20(1.20-1.20) | 0.73 | 0.54 | 0.28 to 0.80 | < 0.95 | 14.29% | 95.24% |
|  | CCL2/MCP-1 | 97.45(79.79-179.3) | 122.2(96.23-175.9) | 0.44 | 0.60 | 0.31 to 0.88 | < 85.78 | 42.86% | 90.48% |
|  | CCL4/MIP-1 beta | 1379(505.8-1697) | 495.4(316.2-923.6) | 0.08 | 0.72 | 0.50 to 0.94 | > 1375.00 | 57.14% | 90.48% |
|  | CXCL8/IL-8 | 1325(429.4-1975) | 697.5(422.2-2333) | 0.69 | 0.55 | 0.29 to 0.80 | > 1285.00 | 57.14% | 71.43% |
|  | CXCL10/IP-10 | 62.32(44.43-83.98) | 54.83(40.51-93.5) | 0.50 | 0.59 | 0.37 to 0.79 | > 59.39 | 71.43% | 57.14% |
|  | G-CSF | 70.75(58.22-82.31) | 51.43(27.07-58.22) | 0.02* | 0.80 | 0.61 to 0.98 | > 61.42 | 71.43% | 85.71% |
|  | GM-CSF | 3.97(3.97-3.97) | 3.97(3.97-3.97) | 1.00 | 0.50 | 0.24 to 0.75 | / | / | / |

Whole blood was collected from newly diagnosed PB leprosy patients and ECs and stimulated overnight with *M. leprae*-specific antigens (ML2044 and LID-1). The concentrations of cytokines and chemokines were determined with Luminex multiplex assays. The AUC and 95% CIs were calculated with ROC analysis. p-values with asterisks indicate significant differences.
