# Supplementary material for: Host immune responses induced by specific Mycobacterium leprae antigens in an overnight whole-blood assay correlate with the diagnosis of paucibacillary leprosy patients in China
Source: PLoS Negl Trop Dis. 2019 Apr 24;13(4):e0007318. doi: 10.1371/journal.pntd.0007318 (PMC6481774; doi:10.1371/journal.pntd.0007318)
Supplement: S5 Table — (DOCX) [file pntd.0007318.s005.docx]

**S5 Table**

| *M. leprae* antigens | Host marker | PB: median (IQR), | TB: median (IQR), | P value | AUC | 95% CI | Cutoff | Sensitivity | Specificity |
| --- | --- | --- | --- | --- | --- | --- | --- | --- | --- |
|  |  | pg/ml | pg/ml |  |  |  |  | % | % |
| ML2044 | TNF-alpha | 8.43(2.84-20.10) | 2.96(0.83-5.53) | 0.04* | 0.77 | 0.56 to 0.97 | > 9.83 | 42.86% | 94.74% |
|  | IL-4 | 46(46-62.79) | 5.24(5.24-21.91) | <0.01* | 0.95 | 0.87 to 1.03 | > 40.81 | 85.71% | 94.74% |
|  | IL-6 | 30.66(15.51-123.80) | 3.27(1.50-5.57) | 0.01* | 0.81 | 0.59 to 1.02 | > 20.67 | 71.43% | 89.47% |
|  | IL-10 | 1.20(0.24-1.20) | 1.20(1.20-1.20) | 0.31 | 0.63 | 0.37 to 0.88 | < 0.31 | 28.57% | 94.74% |
|  | CCL2/MCP-1 | 202.2(147-688.4) | 233.6(110.5-308.5) | 0.40 | 0.61 | 0.37 to 0.84 | > 680.60 | 28.57% | 94.74% |
|  | CCL4/MIP-1 beta | 2414(1278-4291) | 193.9(118.7-337.4) | <0.01* | 0.90 | 0.78 to 1.02 | > 984.80 | 85.71% | 89.47% |
|  | CXCL8/IL-8 | 1060(1060-2040) | 222.7(144.8-553.7) | <0.01* | 0.91 | 0.78 to 1.02 | > 841.50 | 100.00% | 84.21% |
|  | CXCL10/IP-10 | 76.06(64.72-130.5) | 156.5(84.9-237.3) | 0.07 | 0.73 | 0.54 to 0.91 | < 80.48 | 57.14% | 78.95% |
|  | G-CSF | 77.56(56.38-180.5) | 17.33(7.57-56.38) | <0.01* | 0.87 | 0.72 to 1.00 | > 108.50 | 42.86% | 94.74% |
|  | GM-CSF | 3.97(3.97-3.97) | 3.97(1.27-3.97) | 0.50 | 0.59 | 0.34 to 0.83 | > 2.81 | 85.71% | 33.33% |
| LID-1 | TNF-alpha | 2.96(2.31-3.81) | 2.963(1.78-2.96) | 0.54 | 0.58 | 0.33 to 0.82 | > 4.29 | 14.29% | 94.74% |
|  | IL-4 | 12.35(5.24-24.54) | 5.24(5.24-12.35) | 0.15 | 0.68 | 0.41 to 0.94 | > 15.54 | 42.86% | 95.00% |
|  | IL-6 | 21.95(4.1-45.82) | 4.528(2.34-11.9) | 0.09 | 0.72 | 0.47 to 0.96 | > 20.91 | 57.14% | 94.74% |
|  | IL-10 | 1.20(1.20-1.20) | 1.207(1.207-1.207) | 0.90 | 0.52 | 0.26 to 0.76 | < 0.87 | 14.29% | 89.47% |
|  | CCL2/MCP-1 | 97.45(79.79-179.3) | 87.77(57.21-195.7) | 0.60 | 0.57 | 0.33 to 0.79 | > 273.90 | 14.29% | 94.74% |
|  | CCL4/MIP-1 beta | 1379(505.8-1697) | 362.2(89.86-683.1) | 0.02* | 0.80 | 0.60 to 0.99 | > 1233.00 | 57.14% | 94.74% |
|  | CXCL8/IL-8 | 1325(429.4-1975) | 520.6(252.6-776.4) | 0.09 | 0.72 | 0.44 to 0.99 | > 1273.00 | 57.14% | 94.74% |
|  | CXCL10/IP-10 | 62.32(44.43-83.98) | 124.3(82.85-374.4) | 0.01* | 0.83 | 0.66 to 0.98 | < 51.07 | 28.57% | 94.74% |
|  | G-CSF | 70.75(58.22-82.31) | 58.22(27.07-82.31) | 0.22 | 0.66 | 0.44 to 0.86 | > 126.70 | 14.29% | 94.74% |
|  | GM-CSF | 3.97(3.97-3.97) | 3.97(3.97-3.97) | 0.83 | 0.53 | 0.27 to 0.77 | > 2.05 | 100.00% | 5.26% |

Whole blood was collected from newly diagnosed PB leprosy patients and ECs and stimulated overnight with *M. leprae*-specific antigens (ML2044 and LID-1). The concentrations of cytokines and chemokines were determined with Luminex multiplex assays. The AUC and 95% CIs were calculated with ROC analysis. p-values with asterisks indicate significant differences.
