# Supplementary figures and images for: Host immune responses induced by specific Mycobacterium leprae antigens in an overnight whole-blood assay correlate with the diagnosis of paucibacillary leprosy patients in China
Source: PLoS Negl Trop Dis. 2019 Apr 24;13(4):e0007318. doi: 10.1371/journal.pntd.0007318 (PMC6481774; doi:10.1371/journal.pntd.0007318)

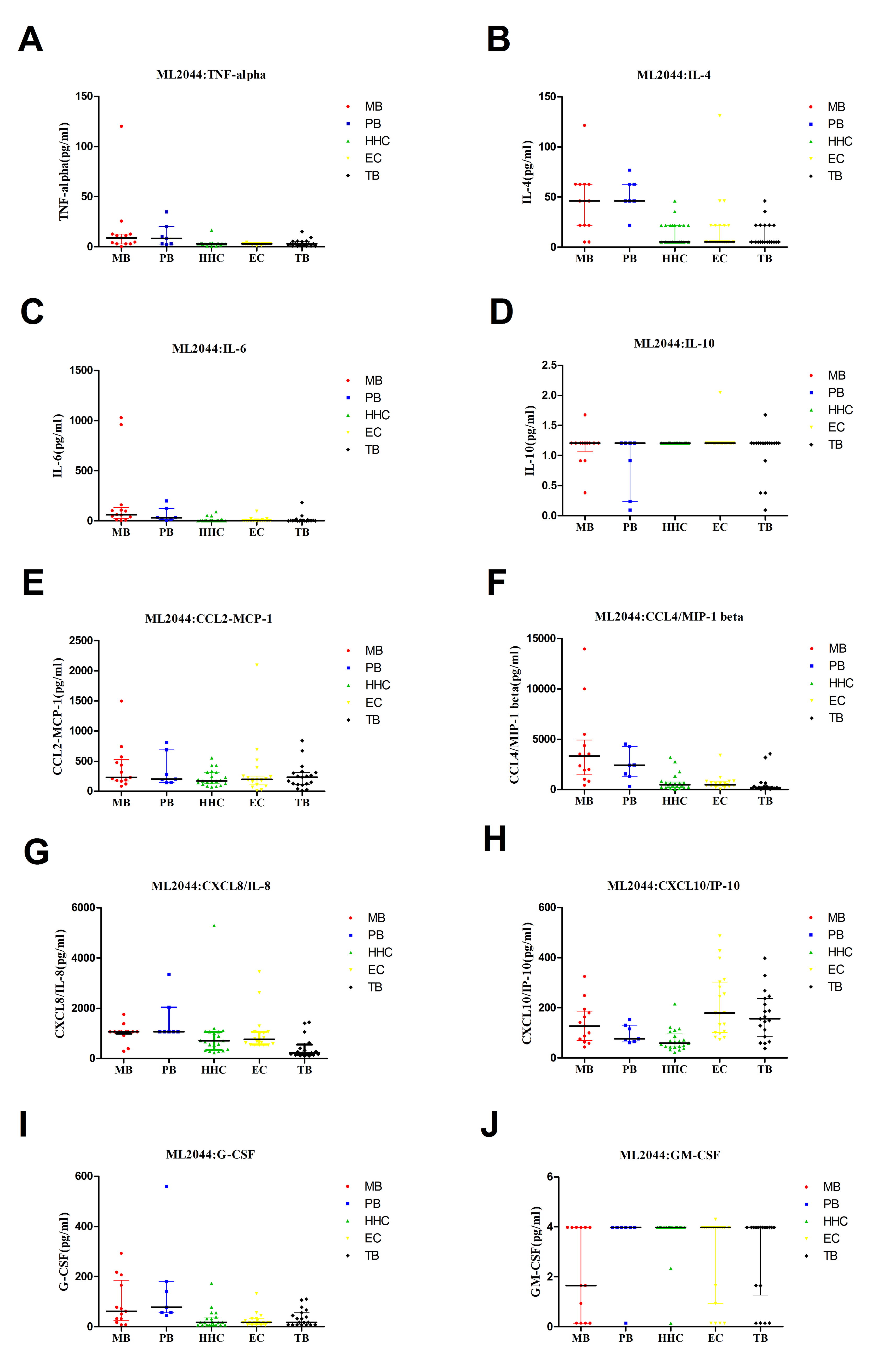

Supplement: S1 Fig — Each dot represents the analyte level of one participant in the study, and horizontal lines represent the median and IQR values. The cytokine and chemokine levels [TNF-α (A), IL-4 (B), IL-6 (C), IL-10 (D), CCL2 (E), CCL4 (F), CXCL8 (G), CXCL10 (H), G-CSF (I) and GM-CSF (J)] obtained in the supernatant after overnight stimulation with the specific M. leprae antigen ML2044 by WBA. (TIF) [file pntd.0007318.s006.tif]

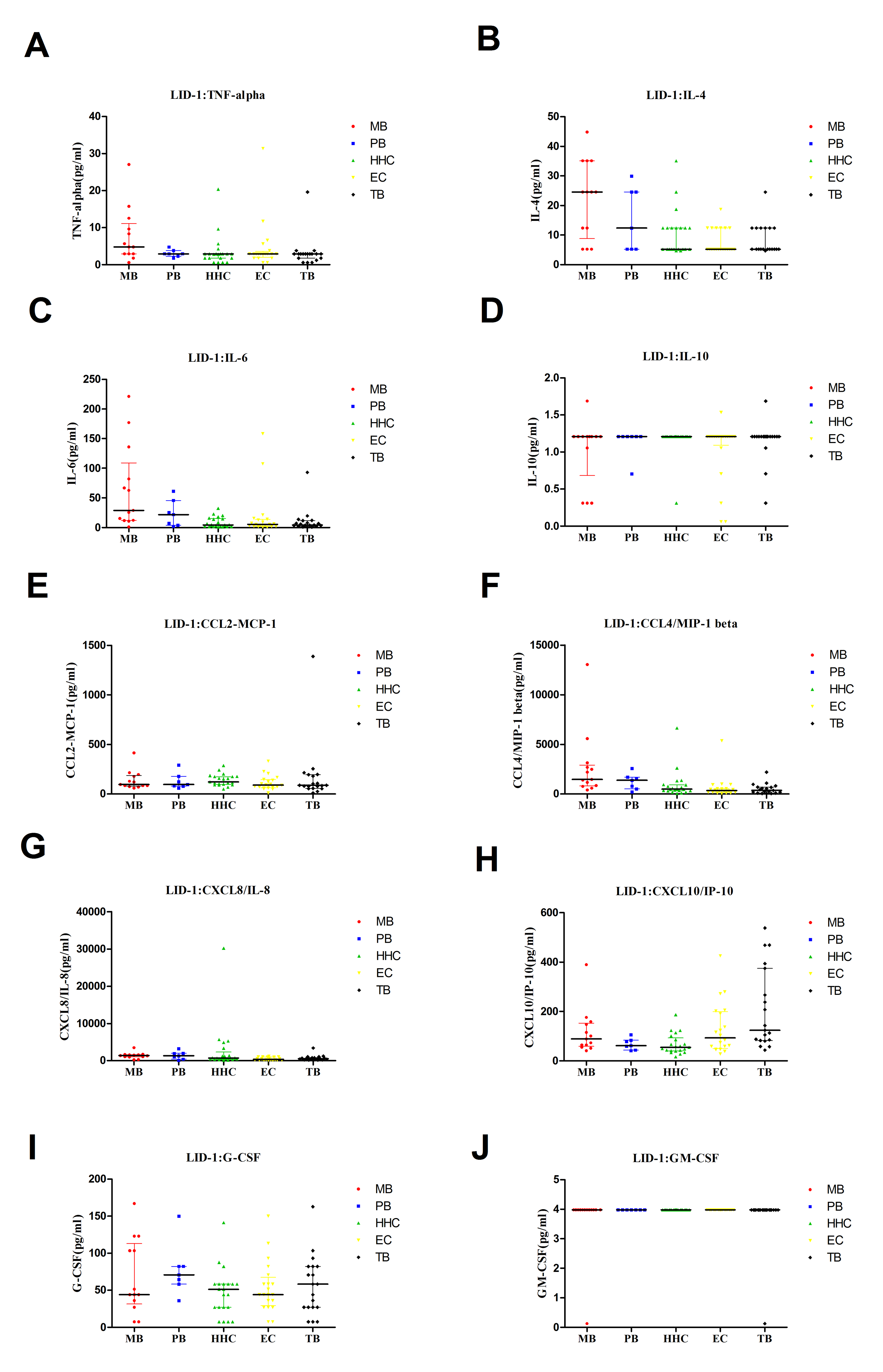

Supplement: S2 Fig — Each dot represents the analyte level of one participant in the study, and horizontal lines represent the median and IQR values. The cytokine and chemokine levels [TNF-α (A), IL-4 (B), IL-6 (C), IL-10 (D), CCL2 (E), CCL4 (F), CXCL8 (G), CXCL10 (H), G-CSF (I) and GM-CSF (J)] obtained in supernatants after overnight stimulation with the specific M. leprae antigen LID-1 by WBA. (TIF) [file pntd.0007318.s007.tif]
