# Supplementary material for: Host immune responses induced by specific Mycobacterium leprae antigens in an overnight whole-blood assay correlate with the diagnosis of paucibacillary leprosy patients in China
Source: PLoS Negl Trop Dis. 2019 Apr 24;13(4):e0007318. doi: 10.1371/journal.pntd.0007318 (PMC6481774; doi:10.1371/journal.pntd.0007318)
Supplement: S3 Fig — Seven PB patients, as defined by the WHO, and 21 HCCs were analyzed. The concentration and the best cutoff were determined for each cytokine or chemokine, as described in S4 Table. The cutoff was used to define whether the concentrations of cytokines and chemokines indicated that the participant was a PB patient or an HHC. Each cytokine or chemokine was used as an independent marker. Prediction of PB patients is shown in dark gray, and prediction of HHCs is shown in light gray for 3 different phenotypic markers. When ≥ 2 phenotypes supported one of the diagnoses, a final diagnosis of either PB (black) or HHC (white) was made. (PDF) [file pntd.0007318.s008.pdf]

ML antigen	ML2044	LID-1	ML2044	Final test result
Host markers	IL-4	G-CSF	CCL4/MIP-1 beta	
PB1				
PB2				
PB3				
PB4				
PB5				
PB6				
PB7				
HHC1				
HHC2				
HHC3				
HHC4				
HHC5				
HHC6				
HHC7				
HHC8				
HHC9				
HHC10				
HHC11				
HHC12				
HHC13				
HHC14				
HHC15				
HHC16				
HHC17				
HHC18				
HHC19				
HHC20				
HHC21				

Marker indicates PB	1 false negative
Marker indicates no PB	1 false positive
Testing indicates no PB	Sensitivity 85.71%
Testing indicates PB	Specificity 100%
