# Supplementary material for: Host immune responses induced by specific Mycobacterium leprae antigens in an overnight whole-blood assay correlate with the diagnosis of paucibacillary leprosy patients in China
Source: PLoS Negl Trop Dis. 2019 Apr 24;13(4):e0007318. doi: 10.1371/journal.pntd.0007318 (PMC6481774; doi:10.1371/journal.pntd.0007318)
Supplement: S4 Fig — Seven PB patients, as defined by the WHO, and 21 HCCs were analyzed. The concentration and the best cutoff were determined for each cytokine or chemokine, as described in S5 Table. The cutoff was used to define whether the concentrations of cytokines and chemokines indicated that the participants was a PB patient or a TB patient. Each cytokine or chemokine was used as an independent marker. Prediction of PB patients is shown in dark gray, and prediction of TB patients is shown in light gray for 3 different phenotypic markers. When ≥ 2 phenotypes supported one of the diagnoses, a final diagnosis of either PB (black) or TB (white) was made. (PDF) [file pntd.0007318.s009.pdf]

ML antigen	ML2044	ML2044	ML2044	Final test result
Host markers	CXCL8	CCL4/MIP-1 beta	IL-4	
PB1				
PB2				
PB3				
PB4				
PB5				
PB6				
PB7				
TB1				
TB2				
TB3				
TB4				
TB5				
TB6				
TB7				
TB8				
TB9				
TB10				
TB11				
TB12				
TB13				
TB14				
TB15				
TB16				
TB17				
TB18				
TB19				

Marker indicates PB	0 false negative
Marker indicates no PB	1 false positive
Testing indicates no PB	Sensitivity 100%
Testing indicates PB	Specificity 94.74%
